# Multiparametric Mapping Magnetic Resonance Imaging of Pancreatic Disease

**DOI:** 10.3389/fphys.2020.00008

**Published:** 2020-02-21

**Authors:** Lixia Wang, Srinivas Gaddam, Nan Wang, Yibin Xie, Zixin Deng, Zhengwei Zhou, Zhaoyang Fan, Tao Jiang, Anthony G. Christodoulou, Fei Han, Simon K. Lo, Ashley M. Wachsman, Andrew Eugene Hendifar, Stephen J. Pandol, Debiao Li

**Affiliations:** ^1^Department of Radiology, Beijing Chaoyang Hospital, Capital Medical University, Beijing, China; ^2^Division of Digestive and Liver Diseases, Cedars-Sinai Medical Center, Los Angeles, CA, United States; ^3^Department of Bioengineering, University of California, Los Angeles, Los Angeles, CA, United States; ^4^Cedars-Sinai Biomedical Imaging Research Institute, Los Angeles, CA, United States; ^5^Department of Nuclear Science and Engineering, Siemens Healthineers, Princeton, NJ, United States; ^6^Department of Gastrointestinal Malignancies, Cedars-Sinai Medical Center, Los Angeles, CA, United States

**Keywords:** pancreatic ductal adenocarcinoma, chronic pancreatitis, magnetic resonance imaging, parametric mapping, T1, T2, ADC

## Abstract

**Background:**

Current magnetic resonance imaging (MRI) of pancreatic disease is qualitative in nature. Quantitative imaging offers several advantages, including increased reproducibility and sensitivity to detect mild or diffuse disease. The role of multiparametric mapping MRI in characterizing various tissue types in pancreatic disease such as chronic pancreatitis (CP) and pancreatic ductal adenocarcinoma (PDAC) has rarely been evaluated.

**Purpose:**

To evaluate the feasibility of multiparametric mapping [T1, T2, and apparent diffusion coefficient (ADC)] in defining tissue characteristics that occur in CP and PDAC to improve disease diagnosis.

**Materials and Methods:**

Pancreatic MRI was performed in 17 patients with PDAC undergoing therapy, 7 patients with CP, and 29 healthy volunteers with no pancreatic disease. T1 modified Look-Locker Inversion Recovery (T1 MOLLI), T2-prepared gradient-echo, and multi-slice single-shot echo-planar diffusion weighted imaging (SS-EPI DWI) sequences were used for data acquisition. Regions of interest (ROIs) of pancreas in PDAC, CP, and control subjects were outlined by an experienced radiologist. One-way analysis of variance (ANOVA) was used to compare the difference between groups and regions of the pancreas, and Tukey tests were used for multiple comparison testing within groups. Receiver operator characteristic (ROC) curves were analyzed, and the areas under the curves (AUCs) were calculated using single parameter and combined parameters, respectively.

**Results:**

T1, T2, and ADC values of the entire pancreas among PDAC, CP, and control subjects; and between upstream and downstream portions of the pancreas in PDAC patients were all significantly different (*p* < 0.05). The AUC values were 0.90 for T1, 0.55 for T2, and 0.71 for ADC for independent prediction of PDAC. By combining T1, T2, and ADC, the AUC value was 0.94 (sensitivity 91.54%, specificity 85.81%, 95% CI: 0.92–0.96), which yielded higher accuracy than any one parameter only (*p* < 0.001).

**Conclusion:**

Multiparametric mapping MRI is feasible for the evaluation of the differences between PDAC, CP, and normal pancreas tissues. The combination of multiple parameters of T1, T2, and ADC provides a higher accuracy than any single parameter alone in tissue characterization of the pancreas.

## Introduction

Pancreatic cancer is the most common pancreatic malignant neoplasm and is the third most common cause of cancer-related deaths from NCI data in 2018. In the United States, pancreatic cancer accounts for about 3% of all cancers in the United States and about 7% of all cancer deaths (American Cancer Society, 2018; Siegel et al., 2018). PDAC is the major subtype of exocrine tumor and constitutes more than 90% of all pancreatic malignancies. Because of the tumor’s unique microenvironment and aggressive nature, PDAC has a relatively poor response to the conventional systemic chemotherapy and poor prognosis, with an overall 5-year survival rate of 8.5% (Yamamoto et al., 2015). Studies found that CP has markedly increased risk for pancreatic cancer (Hao et al., 2017). Five years after diagnosis, CP has a nearly eightfold risk for pancreatic cancer (Kirkegard et al., 2017). Inflammation participates in the development of tumor initiation, progression, treatment response, metastasis, and prognosis (Shi and Xue, 2019). The typical histopathologic features of CP contain acinar cell atrophy, pancreatic fibrosis, leukocyte infiltration, fatty replacement, and distorted and blocked ducts. These findings in CP and PDAC suggest that there are similar radiologic appearances. Further, the upstream pancreas (toward the tail end of the tumor) can have changes of CP due to duct obstruction.

Contrast-enhanced CT was used in PDAC detection, staging, and evaluation of prognosis. A previous study also found that CT radiomics could predict PDAC SMAD4 status and tumor stromal content (Attiyeh et al., 2019). X-ray radiation and iodine allergy are the major risks of CT. Endoscopic ultrasonography (EUS) is the most sensitive non-operative imaging method for the detection of pancreatic cancer and showed to be superior to CT (Luz et al., 2014; Singh and Faulx, 2016). EUS-guided fine needle aspiration (FNB) can achieve cytological information. However, it is invasive and highly operator dependent.

Several studies aimed to quantitatively differentiate PDAC from CP or autoimmune pancreatitis using MR techniques (Park et al., 2009; Fukukura et al., 2012; Yin et al., 2017). DWI plays an important role in the identification of PDAC lesions from the background of pancreatic parenchyma (Ichikawa et al., 2007; Wang et al., 2011; Fukukura et al., 2012; Hayano et al., 2016). With high *b*-value DWI, PDAC lesions can be reliably detected as an increased focal hyper-intensity area (Fukukura et al., 2012).

Hecht et al. (2017) found that the ADC value was significantly lower in tumors with dense fibrosis and may serve as a biomarker of fibrosis architecture. Choi et al. (2016) found that DWI with ADC value was a promising method to differentiate PDAC from mass-forming autoimmune pancreatitis. However, the ADC value only reflects one aspect of the differences between PDAC and CP vs. the normal control pancreas.

T1 and T2 relaxation times are valuable as quantitative parameters to characterize different tissues, especially in myocardial and liver diseases (Apprich et al., 2012; Kali et al., 2015; Blystad et al., 2017; Chen et al., 2017; Vietti Violi et al., 2019). T1 mapping was shown to improve the diagnosis of myocarditis, infarction, iron overload, and amyloidosis (Guo et al., 2009; Karamitsos et al., 2013; Kali et al., 2015). Previous studies found that T1 mapping combined Gd–EOB–DTPA-enhanced MRI can be used to predict the pathologic grading of hepatocellular carcinoma (Cieszanowski et al., 2012; Banerjee et al., 2014; Chen et al., 2017). Recently, Banerjee et al. (2014) and Cassinotto et al. (2015) found that T1 values are strongly correlated with liver fibrosis and liver biopsy in a population of 79 patients. Cieszanowski et al. (2012) demonstrated significantly higher sensitivity and accuracy of T2 relaxation times than ADC values (99.0 and 89.3% vs. 79.0 and 80.9%, respectively) for diagnosing hepatic malignancy. Some studies focused on using the T1 value in the diagnosis and classification of CP and found that T1 could provide quantitative metrics for determining the presence and severity of acinar cell loss and aid in the diagnosis of CP (Tirkes et al., 2017, 2018; Wang et al., 2018). In an animal model of pancreatic cancer, Yin et al. (2017) found that multiparametric MRI was able to characterize pancreatic masses, suggesting that T1, T2, and ADC mapping may have a direct clinical application in patients with PDAC.

In this study, we evaluate the feasibility of non-contrast multiparametric mapping (T1, T2, and ADC) in defining tissue characteristics that occur in CP and PDAC needed for advances in specific diagnosis. The pathological changes in these diseases are complex including changes in cellular density, blood supply, fibrosis, edema, and inflammation. Our pilot study was designed to determine if there are quantitative methods, which can provide specific biomarkers using our novel method non-contract MRI methods based on the hypothesis that a combination of quantitative measures of relaxation time (T1 and T2 values) and ADC values will provide biomarkers that distinguish PDAC, CP, and normal control pancreas.

## Materials and Methods

### Study Population

During the 18-month study period from October, 2017 to April, 2018, patients with PDAC, CP, and normal control pancreas were recruited into this study. All PDAC patients were confirmed by histopathology using tissue obtained by endoscopic ultrasonography (EUS)-guided FNA. The PDAC patients were undergoing neo-adjuvant chemotherapy at the time of the research MRI.

All the patients with CP had diagnosis established by magnetic resonance cholangiopancreatography (MRCP) or endoscopic retrograde cholangiopancreatography (ERCP) with Cambridge classification for CP (Freeny, 1989; Nattermann et al., 1993; Sahai et al., 1998; Tirkes et al., 2018).

The normal control group had no history of acute pancreatitis, CP, diabetes, pancreatic surgery, and no family history of cancer. Patients with pancreatic cystic lesions, benign tumors, or marked pancreatic atrophy or fat degeneration on MRI images were excluded. This prospective study was approved by the local institutional review board. Written informed consents were obtained from all participants.

### Magnetic Resonance Imaging Technique

All subjects were scanned on a 3.0T MR scanner (Biograph mMR, Siemens Healthcare Sector, Erlangen, Germany) with an 18-channel phase array surface coil and were placed head-first, supine position in the magnet. Conventional qualitative sequences and non-contrast quantitative mapping sequences were run for each subject.

Conventional qualitative sequences were transversal T1-weighted three-dimensional volumetric interpolated breath-hold examination (VIBE) with Dixon fat saturation (T1-VIBE-DIXON), and T2 HASTE in transverse, coronal, and sagittal orientations. The parameters of T1-VIBE-DIXON are repetition time (TR) = 4.15 ms; echo time (TE) = 1.39/2.65 ms; flip angle = 9°; field of view (FOV) = 247 × 330 mm; acquisition matrix = 320 × 180; echo train length (ETL) = 2; slice thickness = 3 mm; iPAT acceleration factor = 3. T2 HASTE was performed with the following acquisition parameters: TR = 1,000 ms; TE = 99 ms; flip angle = 105°; FOV = 226 × 330 mm; matrix = 256 × 176; ETL = 109; slice thickness = 5 mm; slice gap = 1 mm; iPAT acceleration factor = 2. MRCP was performed with the following parameters: TR = 8,903 ms; TE = 701 ms; flip angle = 100°; FOV = 300 × 300 mm; acquisition matrix = 384 × 384; ETL = 2; slice thickness = 1 mm; iPAT acceleration factor = 2.

Non-contrast quantitative MRI sequences consisted of 2D MOLLI Trufi with motion correction for T1 mapping, 2D T2-prepared FLASH for T2 mapping, and multi-slice single-shot echo-planar imaging (SS-EPI) for DWI and ADC mapping. T1 MOLLI was acquired using a three-point tool to localize the slice with a single 10-s breath-hold and the following parameters: acquisition window = 280.5 ms; TE = 1.12 ms; echo spacing = 2.7 ms, simulated R–R interval = 1,000 ms; flip angle = 35°; FOV = 390 × 390 mm; matrix = 192 × 144; iPAT acceleration factor = 2. T2-prepared FLASH was acquired, and the slices of T1 MOLLI were copied with a single 10-s breath and the following parameters: acquisition window = 207.4 ms; TR = 3.15 ms, TE = 1.32 ms; echo spacing = 3.1 ms, simulated R–R interval = 1,000 ms, duration of T2 preparations = 0, 30, and 55 ms; flip angle, 12°; FOV, 390 × 390 mm; matrix, 192 × 144; iPAT acceleration factor = 2. For T1 MOLLI and T2-prepared FLASH, three slices located at, above, and below the tumor area were acquired. DWI covered from the dome of the diaphragm to the lower edge of the kidney using the following parameters: *b*-values = 50, 400, and 800 s/mm^2^, TR = 4,500 ms; TE = 47 ms; flip angle, 12°; FOV, 390 × 390 mm; matrix, 192 × 144; ETL = 45; slice thickness = 5 mm; slice gap = 1 mm; iPAT acceleration factor = 2, number of averages = 2 (*b* = 50 s/mm^2^), 4 (*b* = 400 s/mm^2^), and 6 (*b* = 800 s/mm^2^). The parameters of all sequences are listed in Table 1.

**TABLE 1:** Parameters of all MRI sequences used in the study.

Parameters	T1-VIBE- DIXON	T2 HASTE	MRCP	T1 mapping (ms)	T2 mapping (ms)	DWI
Slice thickness (mm)	3	5	1	5	5	6
Gap (mm)	0	1	0	5.5	5.5	1
# Slices/partitions	72	46	80	3	3	50
Repetition time (ms)	4.15	1,000	8,903	2.7	3.15	4,500
Echo time (ms)	1.39/2.65 (OP/IP)	99	701	1.12	1.32	47
Echo train length	2	109	2			45
Acquisition matrix	320 × 180	256 × 176	384 × 384	192 × 126	192 × 144	172 × 132
Flip angle (degree)	9	105	100	35	12	90
FOV (mm)	247 × 330	226 × 330	300 × 300	390 × 390	390 × 390	306 × 399
iPAT acceleration factor	3	2	2	2	2	2
Scan time	18-s single breath-hold	22-s free breathing	10-min free breathing	10 s × 3 breath-hold	10 s × 3 breath-hold	3:50 min free breathing
Additional information			Respiratory trigger		T2 prepared = 0, 30, and 55 ms	b = 50 (2 average), 300 (4 average), 800 (6 average)

### Imaging Analysis

**FIGURE 1:**
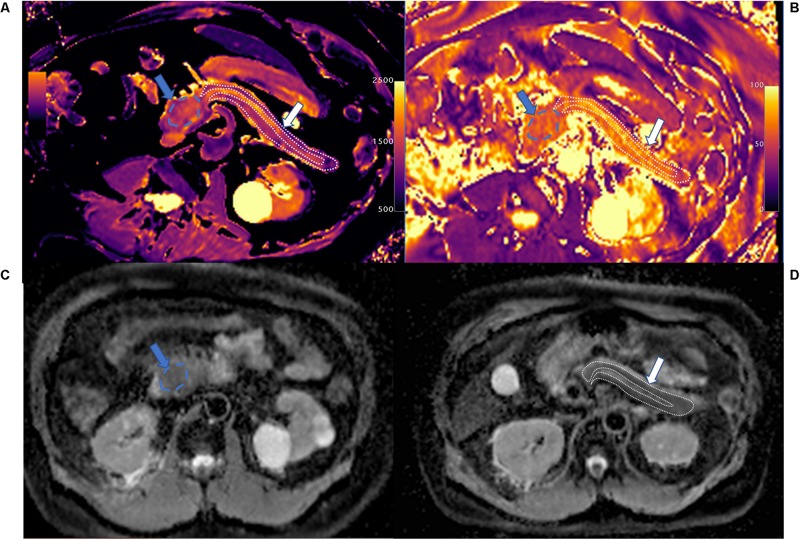
T1 mapping **(A)**, T2 mapping **(B)**, and apparent diffusion coefficient (ADC) maps **(C,D)** of pancreatic ductal adenocarcinoma (PDAC; blue arrow and area) and upstream pancreas (white arrow and area). PDAC shows higher T1 (1,646.7 ± 96.1 ms) and T2 (65.1 ± 9.4 ms) values compared with upstream pancreas, and slightly higher ADC values (1.326 ± 0.098 mm^2^/s) compared with upstream pancreas on ADC maps. Upstream pancreas shows homogeneous lower T1 (1,405.2 ± 149.9 ms), iso-T2 (61.5 ± 9.2 ms), and lower ADC values (1.095 ± 0.261 mm^2^/s). The MPD shows marked dilation and high T1 and ADC values.

**FIGURE 2:**
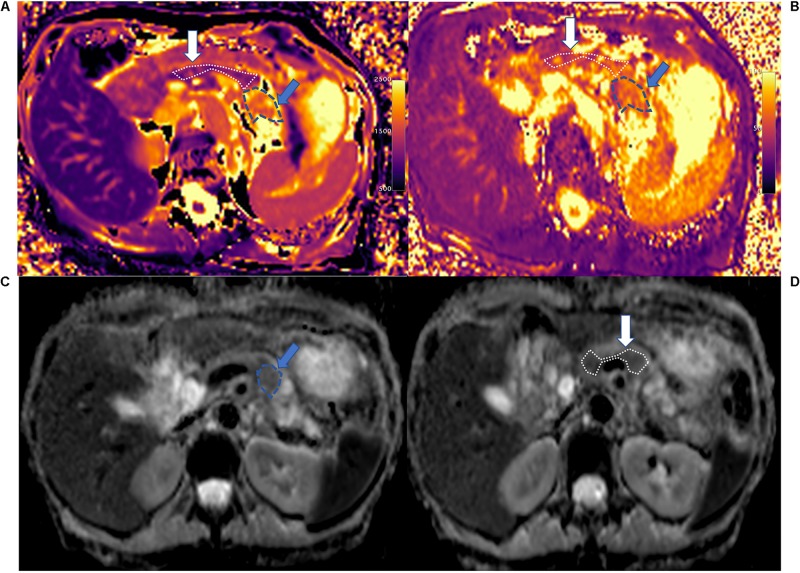
T1 mapping **(A)**, T2 mapping **(B)**, and ADC maps **(C,D)** of PDAC (blue arrow and area) and downstream pancreas (white arrow). PDAC shows higher T1 (1,867.3 ± 163.7 ms), T2 (69.5 ± 6.0 ms), and ADC values (1.857 ± 0.231 mm^2^/s) compared to downstream pancreas. The downstream pancreas shows homogenous T1 (1,266.4 ± 106.9 ms), T2 (62.7 ± 6.3 ms), and ADC (1.315 ± 0.173 mm^2^/s) values. A clear interface is seen between the tumor and downstream pancreas.

**FIGURE 3:**
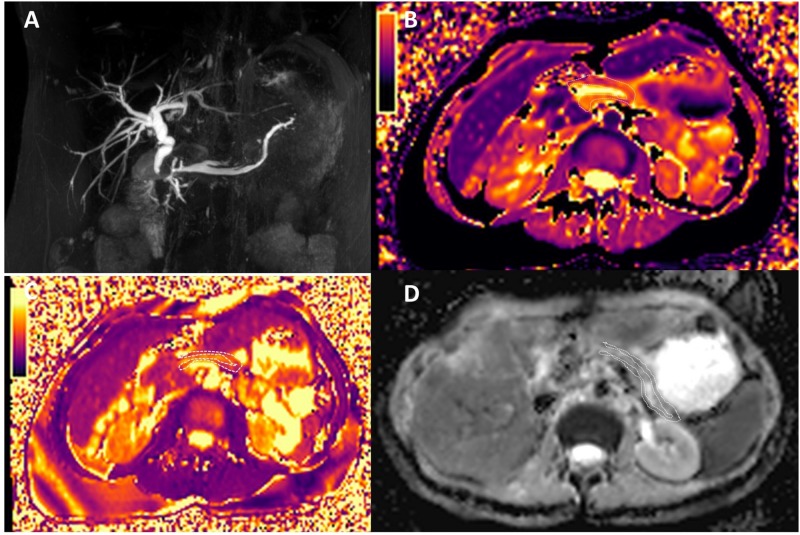
Magnetic resonance cholangiopancreatography **(A)**, T1 mapping **(B)**, T2 mapping **(C)**, and ADC maps **(D)** of chronic pancreatitis (CP). The patient had the history of CP, and the amylase in the blood elevated. MRCP shows dilatation of the major pancreatic duct (MPD) and the branch pancreatic duct; the width of MPD was 4.5 mm. The T1, T2, and ADC of CP were 1,320 ± 220.9 ms, 57.0 ± 9.6 ms, and 1.320 ± 0.162 mm^2^/s, respectively.

**FIGURE 4:**
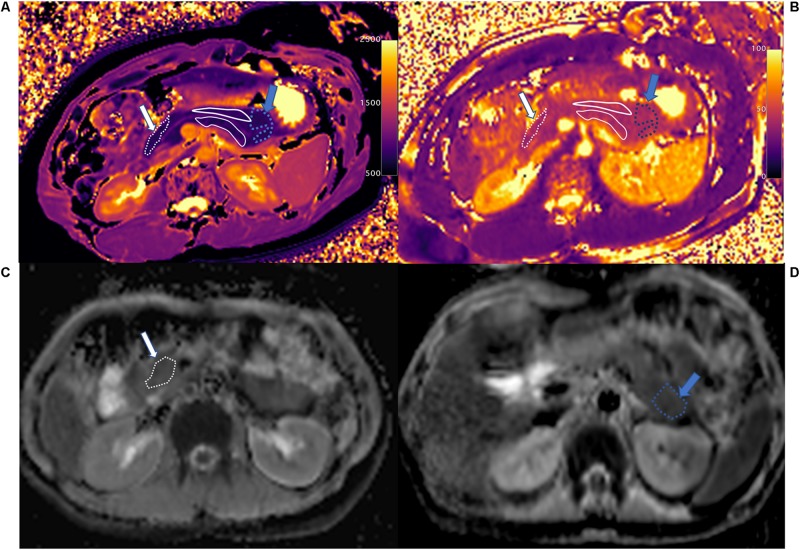
T1 mapping **(A)**, T2 mapping **(B)**, and ADC maps **(C,D)** of normal control pancreatic head (white arrow and area), body (white solid line area), and tail (blue arrow and area). The whole pancreas had the homogenous T1 (head: 798.2 ± 80.5 ms, body: 832.0 ± 83.5 ms, and tail: 835.2 ± 49.7 ms), T2 (head: 45.6 ± 6.5 ms, body: 44.4 ± 4.0 ms, and tail: 45.8 ± 2.2 ms), and ADC (head: 1.206 ± 0.069 mm^2^/s, body: 1.141 ± 0.183 mm^2^/s, and tail: 1.061 ± 0.108 mm^2^/s) values in the head, body, and tail. The main pancreatic duct (MPD) shows clearly on T1 mapping, which differs from the pancreatic head and tail.

T1, T2, and ADC maps of a whole pancreas with PDAC, CP, and normal control pancreas were analyzed and measured along the margin of the pancreas by one radiologist with 20 years of experience in abdominal MRI (LW). We used pixel-wise methods to obtain the mean value of the whole pancreas and avoided areas of necrosis and blood vessels. Tumors were localized with reference to other sequences such as T1-VIBE DIXON, T2 HASTE, DWI images, or the previous contrast-enhanced CT, MRI, or PET/CT within 1 week. In addition, the freehand ROIs (regions of interest) were located within the tumor margin avoiding areas of necrosis. The mean values of tumor, upstream and downstream pancreas (Figures 1, 2), CP (Figure 3), normal pancreatic head, body, and tail (Figure 4) were also obtained based on pixel-wise methods of three slices on T1 and T2 mapping and all the slices on the ADC maps. The tumor’s size was defined as the largest diameter in axial images according to the RECIST 1.1 criteria.

### Statistical Analysis

All the data were analyzed on SPSS v22.0 (Armonk, NY, United States; IBM Corp) and MedCalc (MedCalc for Windows, version 16.2.0.0, Mariakerke, Belgium). Data from the regions of interest were tabulated in a Microsoft Excel worksheet (Microsoft Corporation, Seattle, WA, United States). Descriptive statistics (mean values and the standard deviation) were obtained for the whole pancreas with PDAC, CP, and normal control pancreas, for PDAC mass, upstream and downstream pancreas, and for the head, body, and tail of the normal control pancreas. ANOVA tests were used to compare quantitative parameters between groups, and *post hoc* Tukey tests were used for multiple comparison testing within groups. The value of *p* < 0.05 was considered significant. ROC curve analysis and logistic regression was performed to evaluate the sensitivity and specificity of single parameters and each combination of multiple parameters in the prediction of PDAC after chemotherapy, and the AUCs were calculated. The value of multiparametric mapping over single parameter mapping was determined in MedCalc, using the *Z*-statistic to compare the AUCs of the different ROC curves.

## Results

### Demographics

A total of 53 imaging studies were included (17 patients with PDAC, 7 patients with CP, and 29 volunteers with normal healthy pancreas). Among 17 patients with PDAC, 9 patients were male, and 8 patients were female. The mean age was 65 years old with a range of 46–80 years.

All patients with PDAC were confirmed by FNA and/or surgical specimen. Six lesions were moderately differentiated adenocarcinomas, three lesions were poorly differentiated adenocarcinomas, and the other seven lesions were read as invasive ductal adenocarcinoma, but the grade was not reported.

Based on expert radiologist review of MR images (LW), nine (52.9%) PDACs were located in the pancreatic head, three (17.6%) in the pancreatic neck, two (11.8%) PDACs in the pancreatic body, and three (17.6%) in the pancreatic tail. The median size for all tumors was 3.2 cm, with a range from 1.3 to 6.7 cm.

Among the seven patients (four males and three females) with CP, the mean age was 53 years old with a range of 30–72 years. The mean width of MPD was 4.0 mm with a range from 1.6 to 6.7 mm.

Among the 29 volunteers with normal healthy pancreas, 13 were male and 16 were female. The mean age was 50 years old with a range of 20–72 years.

### Characteristics, Quantification, and Comparison of T1, T2, and ADC Maps

**FIGURE 5:**
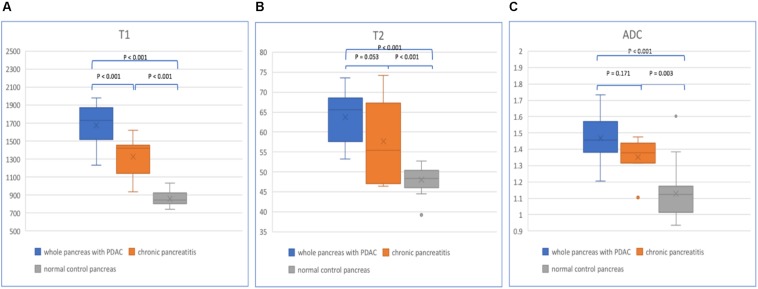
Box plots show T1 **(A)**, T2 **(B)**, and ADC **(C)** values of the whole pancreas with PDAC, CP, and normal control pancreas. *p*-values are listed on the upper box according to one-way analysis of variance (ANOVA).

For the whole pancreas with PDAC, CP, and normal control pancreas, T1, T2, and ADC values are listed in Table 2 (Figure 5). Significant differences were found between the three groups (*p* < 0.001). The whole pancreas with PDAC showed the highest T1 value compared with the CP and normal control pancreas with significant differences found (*p* < 0.001). T1 values for CP were significantly higher than those for the normal pancreas (*p* < 0.001). Significant differences were found when comparing T2 and ADC values of the whole pancreas with PDAC to normal pancreas (*p* < 0.001). In addition, T2 and ADC values of CP had significant differences compared to the normal pancreas (*p* < 0.001 and *p* = 0.003). However, no significant differences were found in the whole pancreas in T2 and ADC between PDAC and CP (*p* = 0.053 and *p* = 0.171).

**TABLE 2:** T1, T2, and ADC values of the whole pancreas with PDAC, chronic pancreatitis (CP), and normal control pancreas.

	T1 value (mean ± SD) (ms)	T2 value (mean ± SD) (ms)	ADC value (mean ± SD) (×10^–3^ mm^2^/s)
Whole pancreas with PDAC	1,675.6 ± 238.1	63.7 ± 6.1	1.519 ± 0.189
Chronic pancreatitis	1,324.0 ± 222.9	57.7 ± 10.7	1.345 ± 0.174
Normal control pancreas	860 ± 70.9	48.0 ± 2.9	1.127 ± 0.158
*p*-Value*	<0.001	<0.001	<0.001

*p-Value is the difference between three groups.

**FIGURE 6:**
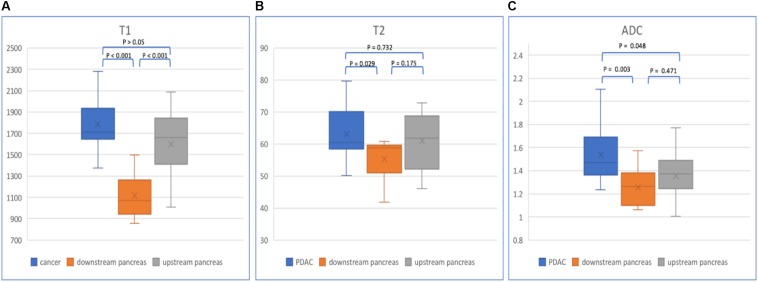
Box plots show T1 **(A)**, T2 **(B)**, and ADC **(C)** values of PDAC, downstream and upstream pancreas. *p*-values are listed on the upper box according to one-way ANOVA.

In patients with PDAC, the T1, T2, and ADC values of mass, upstream and downstream pancreas are listed in Table 3 (Figure 6). The PDAC mass had the highest T1, T2, and ADC values compared with those of the upstream and downstream pancreas. The T1 values showed a significant difference (*p* < 0.001) when comparing the PDAC tumor with the downstream pancreas. The downstream pancreas showed statistically significant differences with the upstream pancreas (*p* < 0.001). No significant differences were found between the PDAC and upstream pancreas (*p* > 0.05). The T2 values were statistically significant (*p* = 0.029) between the PDAC and downstream pancreas, but no significant differences were observed between the PDAC and upstream pancreas (*p* = 0.732), and between the upstream and downstream pancreas (*p* = 0.175). The ADC values were significantly different when comparing PDAC with the upstream (*p* = 0.048) and downstream pancreas (*p* = 0.003). However, no significant difference was found between the upstream and downstream pancreas (*p* = 0.471).

**TABLE 3:** T1, T2, and ADC values of the PDAC, downstream, and upstream.

	T1 value (mean ± SD) (ms)	T2 value (mean ± SD) (ms)	ADC value (mean ± SD) (× 10^–3^ mm^2^/s)
PDAC	1,816.5 ± 208.5	64.9 ± 7.6	1.525 ± 0.243
Downstream pancreas	1,133.1 ± 225.7	54.9 ± 6.9	1.231 ± 0.168
Upstream pancreas	1,598.5 ± 292.0	61.0 ± 9.2	1.355 ± 0.194
*p*-Value*	<0.001	0.037	0.003

*p-Value is the difference between three groups.

**FIGURE 7:**
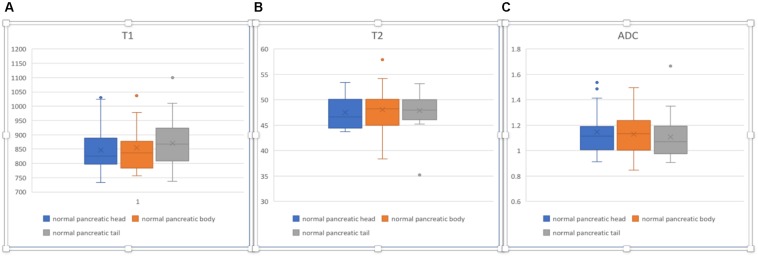
Box plots sho2w T1 **(A)**, T2 **(B)**, and ADC **(C)** values of normal pancreatic head, body, and tail. No significant differences were found according to one-way ANOVA (*p* > 0.05).

In normal pancreas, the head, body, and tail showed relatively homogenous appearances (Figure 7). The T1, T2, and ADC values are listed in Table 4. No significant differences in T1, T2, and ADC values were found between the normal pancreatic head, body, and tail (*p* > 0.05).

**TABLE 4:** T1, T2, and ADC values of normal pancreatic head, body, and tail.

	T1 value (mean ± SD) (ms)	T2 value (mean ± SD) (ms)	ADC value (mean ± SD) (×10^–3^ mm^2^/s)
Normal pancreatic head	846.3 ± 74.6	47.5 ± 2.9	1.146 ± 0.177
Normal pancreatic body	854.6 ± 85.6	48.1 ± 4.2	1.129 ± 0.172
Normal pancreatic tail	870.0 ± 83.2	47.9 ± 3.5	1.108 ± 0.184
*p*-Value*	0.563	0.832	0.802

*p-Value is the difference between three groups.

Comparisons of the parameters between PDAC mass and non-tumor parts of the pancreas in patients with PDAC, CP, and normal pancreas were performed (Table 5). T1 values were significantly different (*p* < 0.001) when comparing PDAC with non-tumor parts of the pancreas, CP, and normal pancreas. T2 and ADC value differences were statistically significant (*p* < 0.05) when comparing PDAC with non-tumor pancreas and normal pancreas. ADC values also shows statistically significant differences (*p* < 0.05) when comparing non-tumor pancreas and CP with normal pancreas. However, no significant differences between CP and non-tumor pancreas were found in T1, T2, and ADC values (*p* > 0.05). T2 and ADC values also showed no significant differences between PDAC and CP (*p* > 0.05).

**TABLE 5:** *p*-Values of one-way analysis of variance (ANOVA) in multiple comparisons of PDAC, non-tumor pancreas, CP, and normal control pancreas.

Comparison groups	T1-value	T2-value	ADC
PDAC vs. non-tumor pancreas	<0.001	0.026	0.003
PDAC vs. chronic pancreatitis	<0.001	0.624	0.117
PDAC vs. normal control pancreas	<0.001	<0.001	<0.001
Non-tumor pancreas vs. chronic pancreatitis	0.636	0.720	0.945
Non-tumor pancreas vs. normal control pancreas	<0.001	0.266	0.015
Chronic pancreatitis vs. normal control pancreas	<0.001	0.071	0.025
Upstream pancreas vs. normal control pancreas	<0.001	<0.001	0.003
Downstream pancreas vs. normal control pancreas	<0.001	0.003	0.435

### Differentiation of PDAC Mass From Non-tumor Pancreas

**FIGURE 8:**
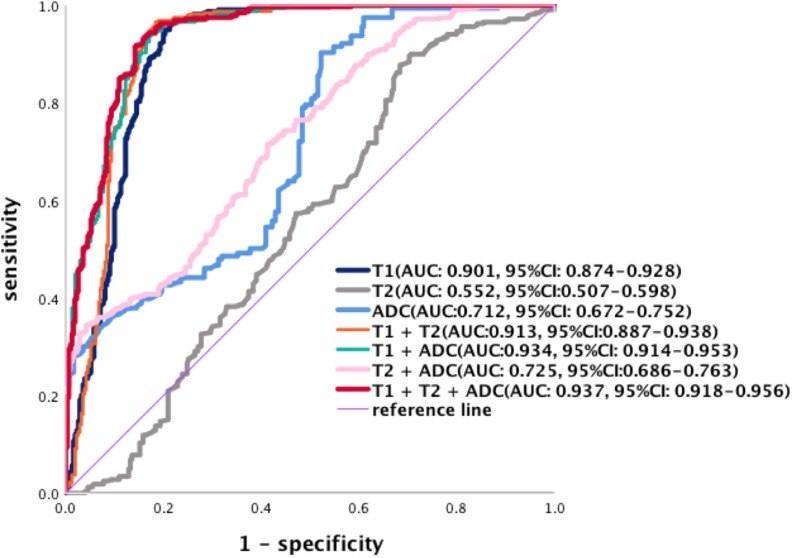
Receiver operating characteristic curves of the diagnostic performance of T1, T2, and ADC values and the different combinations of parameters in the differentiation of PDAC. The AUCs (95% CI) are T1 [0.901 (0.874–0.928)], T2 [0.552 (0.507–0.598)], and ADC values [0.712 (0.672–0.752)]. Combination of two parameters, the AUCs (95% CI) of T1 + ADC [0.934 (0.914–0.953)], T1 + T2 [0.913 (0.887–0.938)], and T2 + ADC [0.725 (0.686–0.763)]. The AUCs (95% CI) for the combination of T1, T2, and ADC are [0.937 (0.918–0.956)].

Evaluation by ROC curves and the AUCs for each parameter and combinations of parameters was performed (Figure 8). Using single parameters, T1 values yielded the greatest AUC (95% CI) [0.901 (0.874–0.928)] compared to T2 [0.552 (0.507–0.598)] and ADC [0.712 (0.672–0.752)]. Based on the ROC analysis, the cutoff values of T1, T2, and ADC for the differentiation of PDAC mass undergoing chemotherapy from non-tumor pancreas were 1,494.0, 59.3 ms, and 1.201 × 10^–3^ mm^2^/s, respectively. The sensitivity and specificity were for T1 (96.55 and 78.39%), T2 (87.77 and 31.61%), and ADC values (91.60 and 47.74%) were calculated. When combining two parameters, the highest AUC (95% CI) was obtained when combining T1 and ADC [0.934 (0.914–0.953)] compared to a combination of T1 and T2 [0.913 (0.887–0.938)], or a combination of T2 and ADC [0.725 (0.686–0.763)]. When T1, T2, and ADC were combined, the AUC (95% CI) was 0.937 (0.918–0.956) with a sensitivity of 91.54% and a specificity of 85.81%, and this was significantly higher than that using any single parameter (*p* < 0.001). There was no statistical difference between a combination of T1, T2, and ADC vs. T1 and ADC (*p* = 0.158). The sensitivity and specificity of each curve is listed in Table 6.

**TABLE 6:** Sensitivity and specificity of each single parameter and combinations of multi-parameters.

	Sensitivity (%)	Specificity (%)
T1	96.55	78.39
T2	87.77	31.61
ADC	91.60	47.74
T1 + T2	96.55	81.61
T1 + ADC	93.73	82.90
T2 + ADC	34.5	95.8
T1 + T2 + ADC	91.54	85.81

## Discussion

In this study, significant differences were found when defining the tissue characteristics of the whole pancreas with PDAC, CP, and normal control pancreas, and comparing PDAC mass, upstream and downstream pancreas using T1, T2, and ADC values. The differences between the measured T1, T2, and ADC values in whole pancreas with PDAC, CP, and normal pancreas may reflect the development of CP to PDAC in some extent and may provide a new follow-up method of CP. The difference between non-tumor pancreas, especially upstream pancreas and downstream pancreas, and in normal control pancreas was pathologically proven to correlate with the inflammatory pathological change, which plays an important role on the tumor response of chemotherapy, recurrence, and 5-year survival rate (Hayano et al., 2016). Furthermore, we found that a combination of T1, T2, and ADC values was superior in characterizing tissue properties when compared to any single parameter.

Normal healthy pancreas has the lowest T1, T2, and ADC values compared to the PDAC group and CP group. No significant differences were found between the pancreatic head, body, and tail (*p* > 0.05). Normal healthy pancreas has a short T1 value compared with other abdominal organs due to the presence of a high amount of acinar protein and rough endoplasmic reticulum in the pancreatic cells (Tirkes et al., 2017; Noda et al., 2019). In our study, the mean ADC value showed a slight trend of reduction from head to tail (head 1.146 × 10^–3^ mm^2^/s, body 1.129 × 10^–3^ mm^2^/s, and tail 1.108 × 10^–3^ mm^2^/s) due to the heterogeneity of pancreatic tissue composition, and this is consistent with the prior report (Schoennagel et al., 2011).

Chronic pancreatitis results in irreversible pancreatic structural damage, and the pathological changes include pancreatic calcification, chronic inflammation, and fibrosis, which correlate with an increase in T1 signal intensity in pancreatic parenchyma (Yoon et al., 2016). An increased T1 value was demonstrated with progressive disease (normal controls 865 ± 220 ms vs. mild CP 1,075 ± 221 ms vs. severe CP 1,350 ± 139 ms, *p* < 0.0001) (Wang et al., 2018) in a previous study. In our study, the T1 value in CP was markedly higher than that of the normal control pancreas (*p* < 0.001) and lower than that of the whole pancreas with PDAC (*p* < 0.001). CP also has longer T2 and higher ADC than those of the normal control pancreas, which may be related with edema, fibrosis, gland atrophy, and fat infiltration.

The T1, T2, and ADC values of the whole pancreas with PDAC were highest compared with the other two groups for the malignant tumor, fibrosis, and COP in the upstream pancreas and TACP around the tumor. The differences in T1 values were statistically significant within the whole pancreas with PDAC, CP, and normal control pancreas (*p* < 0.001). However, T2 and ADC values showed no significant differences between the whole pancreas with PDAC and CP for the pathological changes of CP and upstream pancreas.

Pancreatic ductal adenocarcinoma tends to obstruct MPD, which results in COP, fibrosis, and atrophy of the upstream pancreas (Balkwill and Mantovani, 2001; Coussens and Werb, 2002; Mantovani et al., 2008). COP may be caused by the tumor obstruction of MPD, which leads to increased intraductal pressure and damage to the duct membrane or rupture of the secondary ducts resulting in interstitial extravasation of the activated pancreatic enzymes, the recurrence of inflammation, and interstitial damage results in fibrosis hyperplasia and CP. Another hypothesis is that tumor cells may secrete plasminogen-activating enzymes, which may, in turn, activate trypsinogen-inducing auto-digestion. In some patients, tumor can arise from pre-existing CP (Leal and Liby, 2018). On the other hand, the downstream pancreas may be less influenced than the upstream pancreas.

Significant differences (*p* < 0.05) when comparing non-tumor pancreas with PDAC mass, upstream pancreas with downstream pancreas, and downstream pancreas with normal control pancreas can be interpreted by COP or TACP (Imamura et al., 1995). TACP was found in the adjacent parenchyma of the PDAC mass. In addition, this can be used to interpret why T1 and T2 values in the downstream pancreas showed significant differences when compared with the normal control pancreas (*p* < 0.05). The results also proved that the inflammatory changes and fibrosis in the upstream pancreas are markedly more severe than the downstream pancreas (Bali et al., 2011).

Receiver operator characteristic analysis showed that for the single parameters, the T1 value is the most accurate in differentiating the tumor and non-tumor area compared with that of the T2 value and ADC value (*p* < 0.001). The T2 and ADC values have some limitations when a large amount of fibrosis and the change in tumor cellular density are present in the tumor and upstream pancreas. In our study, we found that if three parameters were combined, the AUC was statistically significantly higher compared with any single parameter and two combined parameters (*p* < 0.05) with the exception of the combined T1 and ADC values (*p* = 0.158). Therefore, it appears that the T1 value is critical in tissue characterization, while the ADC value can provide additional helpful information but has lower sensitivity and specificity than the T1 value. Based on our data, the T2 value only provides minimal information in tissue characterization.

Our study has some limitations. First, the relatively small numbers of patients may affect the statistical outcomes. These findings will need to be validated in larger studies. Second, patients were not sub-grouped according to the grade of cancer; therefore, the T1, T2, and ADC values may show wide variability. Subgrouping of patients was not possible due to the small pilot nature of this study. Third, most patients with PDAC underwent chemotherapy at the time of study. This may have altered the tumor characteristics. We found from our research group experience that patients who were treatment naïve are difficult to recruit. Fourth, the ROIs were obtained according to the previous imaging data and other non-contrast MR images, and there was an interval between the MR scan and surgery. The tumor margin could not be validated immediately, so we selected a relatively smaller ROI within the tumor, avoiding the necrosis area. Fifth, T1 and T2 mapping only included three slices because we used the T1 MOLLI sequence. This would not reflect the global features of the tumor. Future studies should recruit patients prior to initiation of treatment and over the course of the treatment so that there are independent predictors of prognosis in the responder and non-responder. Finally, the T1, T2, and ADC maps had different resolutions at the time of image acquisition, which required retrospective calibration to keep the pixel numbers consistent. In future studies, we will keep the same T1, T2, and DWI image resolutions and continuous or three-dimensional T1 and T2 mapping sequences in imaging acquisition to decrease the bias. For the multiparametric mapping, which can demonstrate the differences of PDAC mass undergoing chemotherapy, upstream and downstream pancreas, we believe that it can be used as a biomarker to predict tumor or longitudinal follow-up after chemotherapy.

## Conclusion

**FIGURE 9:**
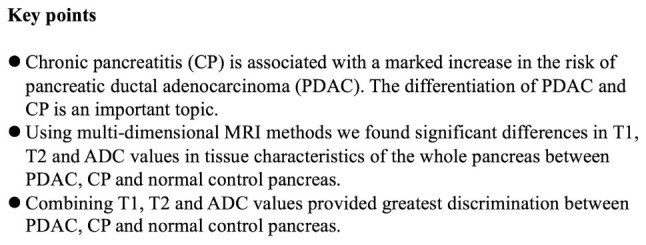
The key points about this article.

In conclusion, multiparametric mapping is feasible for the evaluation of the differences between PDAC, CP, and normal pancreas tissue. The combination of multiple parameters of T1, T2, and ADC provides a higher accuracy compared to the result with any single parameter in tissue characterization of the pancreas (the main points of this article are listed in Figure 9).

## Data Availability Statement

All datasets generated for this study are included in the article/supplementary material.

## Ethics Statement

The studies involving human participants were reviewed and approved by the Cedars-Sinai Institutional Review Board. The patients/participants provided their written informed consent to participate in this study. Written informed consent was obtained from the individual(s) for the publication of any potentially identifiable images or data included in this article.

## Author Contributions

LW, DL, and SP conceived and designed the study. LW and NW analyzed and interpreted the data. NW, ZD, ZZ, and AH collected the data. LW and SG wrote the manuscript. SG, ZF, TJ, AC, FH, SL, and AW critically revised the manuscript. DL and SP approved the final version of the manuscript. LW and YX contributed to statistical analysis.

## Conflict of Interest

FH was employed by company Siemens Healthineers. The remaining authors declare that the research was conducted in the absence of any commercial or financial relationships that could be construed as a potential conflict of interest.

## Abbreviations

ADC: apparent diffusion coefficient
ANOVA: one-way analysis of variance
AUC: area under curve
COP: chronic obstructed pancreatitis
CP: chronic pancreatitis
DWI: diffusion weighted imaging
MPD: main pancreatic duct
MRI: magnetic resonance imaging
PDAC: pancreatic ductal adenocarcinoma
ROC: receiver operator characteristic
T1 MOLLI: T1 modified Look-Locker Inversion Recovery
T1-VIBE-DIXON: T1-weighted volumetric interpolated breath-hold examination
T2 HASTE: T2 half-Fourier acquisition single-shot turbo spin–echo sequence
TACP: tumor-associated chronic pancreatitis.
